# Mono(2-ethylhexyl) Phthalate Disrupts Mitochondrial Function, Dynamics and Biogenesis in Human Trophoblast Cells at Human Exposure Range Concentrations

**DOI:** 10.3390/toxics13090770

**Published:** 2025-09-11

**Authors:** Luis Daniel Martínez-Razo, Nadia Alejandra Rivero-Segura, Ericka Karol Pamela Almeida-Aguirre, Ismael Mancilla-Herrera, Ruth Rincón-Heredia, Alejandra Martínez-Ibarra, Marco Cerbón

**Affiliations:** 1 Facultad de Química, Universidad Nacional Autónoma de México, Mexico City 04510, Mexico; ludamara3009@comunidad.unam.mx (L.D.M.-R.); karol.almeida.aguirre@gmail.com (E.K.P.A.-A.); alejandra_martinez@hotmail.es (A.M.-I.); 2 Servicio de Genética, Hospital General de México Dr. Eduardo Liceaga, Mexico City 06720, Mexico; 3 Dirección de Investigación, Instituto Nacional de Geriatría, Mexico City 10200, Mexico; nrivero@inger.gob.mx; 4 Departamento de Infectología e Inmunología, Instituto Nacional de Perinatología Isidro Espinosa de los Reyes, Mexico City 11000, Mexico; ismael.mancilla@quimica.unam.mx; 5 Unidad de Imagenología, Instituto de Fisiología Celular, Universidad Nacional Autónoma de México, Mexico City 04500, Mexico; rrincon@ifc.unam.mx

**Keywords:** MEHP, trophoblast, mitochondrial dysfunction, mitochondrial dynamics, mitochondrial biogenesis

## Abstract

Mono(2-ethylhexyl) phthalate (MEHP), a bioactive metabolite of di(2-ethylhexyl) phthalate (DEHP), has been detected in the placenta and urine of pregnant women and is linked to adverse pregnancy outcomes. However, its effects on mitochondrial homeostasis in trophoblast cells remain incompletely understood. This study examined the impact of MEHP (0.5–200 µM) on mitochondrial function, dynamics, and biogenesis in human HTR-8/SVneo trophoblast cells. MEHP (≥5 µM) reduced MTT conversion without compromising membrane integrity, suggesting early metabolic or redox imbalance. A dose-dependent loss of mitochondrial membrane potential was observed, with increased reactive oxygen species (ROS) generation only at 200 µM. MEHP modulated the expression of mitochondrial dynamics genes, with a more pronounced mitofusin 1 (*MFN1*) induction at low doses and increased mitochondrial DNA content, suggesting a compensatory response to mild stress. Conversely, high doses more strongly induced fission and mitochondrial 1 (*FIS1*) expression, suggesting mitochondrial fragmentation. Both concentrations induced the expression of the mitochondrial biogenesis regulators peroxisome proliferator-activated receptor gamma coactivator 1 alpha (PGC-1α) and nuclear factor erythroid 2–related factor 2 (Nrf2), while sirtuin 1 (SIRT1) expression and activity declined progressively with dose. These results demonstrate that MEHP disrupts mitochondrial homeostasis in trophoblast cells at concentrations spanning the estimated human exposure range. The dose-dependent effects, from adaptive responses to overt dysfunction, may help explain the associations between MEHP exposure and placental pathology observed in epidemiological studies.

## 1. Introduction

Di(2-ethylhexyl) phthalate (DEHP) is one of the most widely used plasticizers in the plastics industry, employed in various applications, including food and beverage packaging, toys, vehicles, cosmetics, pharmaceuticals, medical devices, and PVC pipes [1]. Although phthalates have been used in industry since the 1920s, their increased use over time has evidenced that humans can ingest them by consuming various products packaged in plastics that employ phthalates in their manufacture [2,3]. Consequently, regulatory frameworks have been established to control the use of these compounds in specific products, including toys, medical devices, cosmetics, and food. However, the implementation of regulatory health policies in this field is limited, as only a few regions have adopted such measures, including the United States, the European Union, and China, which have identified DEHP as a priority hazardous pollutant. Nevertheless, significant disparities remain regarding the limits and products in which they are permitted, which underscores the persistence of this global public health problem, especially in regions without regulatory oversight [3]. In the human body, most phthalates are rapidly converted into monoesters, such as mono(2-ethylhexyl) phthalate (MEHP), a metabolite of DEHP, and are excreted within 24–48 h [4,5]. Despite the extensive evaluation of these urinary metabolites in various populations, the results show significant variability. For instance, the mean concentrations of MEHP in human urine range from 3 to 10 µg/L. However, certain studies have documented levels as high as 47,071 µg/L, which, though exceptionally elevated, underscores the heterogeneity of MEHP exposure across populations and the potential influence of environmental, cultural, and social factors, as previously suggested [6,7,8,9,10].

It has been demonstrated that exposure to DEHP, and particularly its metabolite MEHP, is associated with an elevated risk of developing various diseases, including several types of cancer and metabolic and reproductive disorders [11,12,13,14]. Both compounds belong to the category of endocrine-disrupting chemicals (EDCs), which can compromise hormone regulation in ingesting individuals. However, the threat posed by these chemicals is more significant when they are consumed during specific stages of life [14]. For instance, the exposure to EDCs during pregnancy has been demonstrated to exert a detrimental influence on maternal health and fetal development. Particularly, the exposure to DEHP/MEHP has been associated with pregnancy-related pathologies, including preeclampsia, intrauterine growth restriction (IUGR), gestational diabetes mellitus (GDM), and preterm delivery. These conditions are characterized by placental dysfunction, specifically the extravillous cytotrophoblast (EVT) [6,15,16,17,18].

A growing number of studies have described the effect of DEHP/MEHP on the trophoblast. For instance, MEHP (90 and 180 μM) increased reactive oxygen species (ROS) generation, mRNA expression of prostaglandin-endoperoxide synthase 2 (*PTGS2*), an enzyme important for the synthesis of prostaglandins implicated in the initiation of labor; oxidative DNA damage, and caspase 3/7 activity in an in vitro model of human EVT cells (HTR-8/SVneo cells) [19]. A subsequent study showed that this increased ROS production leads to apoptosis at MEHP high concentrations through microRNA-mediated mechanisms in HTR-8/SVneo cells [20]. Other studies have explored broader effects of MEHP on the transcriptome and metabolome of HTR-8/SVneo cells, showing alterations in nuclear hormonal pathways and amino acids, pyrimidine, and glutathione metabolism, respectively [21,22]. In addition, our research group reported high levels of MEHP (11,855 µg/L) in urine from women with GDM and an association with the expression of miR-29a-3p, an important microRNA in the regulation of glucose metabolism and fatty acid oxidation targeting genes such as *PPARGC1A* [6]. Altogether, this evidence suggests that MEHP may be compromising metabolic function, specifically at the mitochondrial level, as oxidative damage has also been reported.

Mitochondrial function is crucial for trophoblast physiology, as it conducts high metabolic demands associated with proliferation, invasion, and differentiation during early placental development. These processes depend on adequate mitochondrial ATP production, redox balance, and regulation of apoptosis, all of which are essential for proper remodeling of maternal tissues and successful establishment of the maternal–fetal interface [23,24,25]. Impaired mitochondrial metabolism in trophoblasts may therefore compromise placental development and contribute to the etiology of pregnancy disorders [24,26,27,28]. In this sense, Meruvu et al. (2024) reported that MEHP concentrations ranging from 50 to 360 μM induce alterations in mitochondrial function in HTR-8/SVneo cells, such as hypoxia, disrupted mitochondrial ATP generation and membrane potential, as well as lowered mtDNA copy number and inhibited expression of electron transport chain subunits [29]. While informative, the study examines MEHP concentrations within the upper range of exposure, without addressing lower levels reported in human tissues. To better reflect physiologically relevant conditions, we focused on a broader concentration range (0.5–200 μM) corresponding to levels measured in placental tissue in a CANDLE Study (Conditions Affecting Neurocognitive Development and Learning in Early childhood) [30] and levels estimated in maternal plasma from a highly exposed population [6]. It is noteworthy that the levels of MEHP in the placenta do not exhibit significant disparities when compared to plasma estimates within a population that is extensively exposed to MEHP; in both instances, the levels are observed within the micromolar range. This observation suggests the possibility that the placenta may serve as a repository for MEHP, which could potentially exert deleterious effects on maternal and fetal health. Hence, the present study aimed to characterize the effects of MEHP on mitochondrial homeostasis, including activity, biogenesis, and dynamics (fusion and fission) in HTR-8/SVneo trophoblast cells. This approach may help explain the molecular mechanisms underlying the associations between MEHP exposure and pregnancy disorders related to placental dysfunction previously reported.

## 2. Materials and Methods

### 2.1. Cell Culture and Treatment

HTR-8/Svneo cells (#CRL-3271, ATCC, Manassas, VA, USA) were cultured in RPMI-1640 medium (#30-2001, ATCC, Manassas, VA, USA) supplemented with fetal bovine serum (FBS) 5% (#S1620, Biowest, Nuaillé, France) and 1% penicillin/streptomycin (#15240062, Gibco, Thermo Fisher Scientific, Inc., Waltham, MA, USA) at 37 °C in a humidified incubator with 5% CO_2_. Cells grew to a confluence of 60–70% before being treated for 24 or 48 h.

### 2.2. Chemicals and Reagents

MEHP (97%; CAS 4376-20-9) was purchased from Sigma-Aldrich (#796832, Oakville, ON, Canada) and dissolved in DMSO at stock concentrations to achieve final concentrations ranging from 0.5 to 200 μM for treatments. The final DMSO concentration was 0.125 % for all treatments and vehicle-treated as controls. MTT (3-(4,5-dimethylthiazol-2-yl)-2,5-diphenyltetrazolium; CAS 298-93-1; #M6494, Thermo-Fisher Scientific, Waltham, MA, USA) was dissolved in PBS at a stock concentration of 2.5 mg/mL for the cell viability assay. JC-1 (5,5″,6,6″-tetrachloro-1,1″,3,3″-tetraethylbenzimidazolylcarbocyanineiodide; #T3168; Thermo-Fisher Scientific, Waltham, MA, USA) was dissolved at a stock concentration in DMSO. All chemicals were stored at −20 °C prior to use.

### 2.3. Cell Viability Assays

#### 2.3.1. Flow Cytometric Detection of Cell Membrane Damage (Fixable Viability Dye)

To analyze cell viability, cells were incubated with Fixable Viability Dye eFluor506 (FVD, eBiosciences, Carlsbad, CA, USA) and then fixed as previously described [31]. Cells were analyzed in a FACS Aria III flow cytometer using the DIVA software V.6.1.3 (BD Biosciences, Franklin Lakes, NJ, USA), counting at least 10,000 events. Cell viability by determining cytoplasmic membrane integrity was evaluated 24 and 48 h after treatment.

#### 2.3.2. MTT Assay

Mitochondrial activity was indirectly evaluated by the MTT assay. HTR-8/Svneo cells were seeded on 96-well plates at 8000 cells/well. After 24 h, cells were treated with either DMSO or MEHP. MTT reduction was evaluated 24 and 48 h later, as previously described [32]. The absorbance of the formazan salt was read at 570 nm on a microplate BioTek Epoch Microplate Spectrophotometer (Agilent Technologies, Santa Clara, CA, USA). All determinations were registered from five independent experiments.

### 2.4. ROS Quantification Assay

Cellular ROS was evaluated using the DCFDA/H2DCFDA—Cellular ROS Assay Kit (ab113851, Abcam, Cambridge, UK). The assay was performed following the manufacturer’s instructions for adherent cells treated for 48 h with either DMSO or MEHP. Images were captured using an Olympus IX-71 inverted microscope with an original magnification of 20x. Four different fields were captured from each of the three replicates for all conditions. Quantitative data were obtained by reading at 485/535 nm on a microplate BioTek Epoch Microplate Spectrophotometer (Agilent Technologies, Santa Clara, CA, USA).

### 2.5. Mitochondrial Membrane Potential Determination

Mitochondrial membrane potential was measured with the JC-1 fluorescent marker according to the methodology reported in [33]. Briefly, we seeded 350 × 103 HTR-8/Svneo cells on glass coverslips. Cells were exposed to MEHP at 5 μM or 200 μM for 48h at standard culture conditions (37 °C in a humidified incubator with 5% CO_2_). Then, cells were incubated with JC-1 (100 nM) for 30 min in the dark at standard culture conditions. As a positive control of mitochondrial dysfunction, 1 μM of FCCP (Sigma-Aldrich, St. Louis, MO, USA) was incubated for 5 min. FCCP is a protonophore, a chemical compound that can transport protons across the mitochondrial membrane, leading to mitochondrial uncoupling due to disruption of oxidative phosphorylation. FCCP has been used as a positive control in the JC-1 assay because a loss of mitochondrial membrane potential results in a drastic decrease in the 530/590 fluorescence ratio. Afterwards, coverslips were transferred to a chamber with Lockey’s buffer at 37 °C and observed under a confocal microscope (Zeiss LSM800) using the argon laser, at Unidad de Imagenología del Instituto de Fisiología Celular, UNAM. Images were captured from five different fields in each condition and analyzed as previously described, calculating the 530/590 nm ratio [33].

### 2.6. Mitochondrial DNA Content

The relative copy number of mtDNA was determined based on the ratio of mtDNA to nuclear DNA (nDNA) by quantitative real-time PCR (qPCR). Mitochondrially encoded NADH dehydrogenase 1 (*MT-ND1*) was used as an mtDNA target, while a sequence from the *GAPDH* promoter was used as a nuclear DNA internal control. Primer sequences were previously reported by Liu et al., 2021 [34]. Total DNA was extracted from HTR-8/Svneo (*n* = 3) cells using the Wizard^®^ Genomic DNA Purification Kit (Promega, Madison, WI, USA) following the manufacturer’s instructions. qPCR was performed using Power Up SYBR Green (Applied Biosystems, Waltham, CA, USA) following standard conditions using a CFX96 thermocycler (Bio-Rad, Berkeley, Hercules, CA, USA). The ΔΔCt method was used to calculate the mtDNA/nDNA ratio.

### 2.7. RNA Isolation and RT-qPCR

Total RNA was isolated using the RNeasy Plus Mini Kit (Qiagen, Hilden, Germany) according to the manufacturer’s instructions. RNA quantification was performed using Nanodrop One (Thermo Fisher Scientific, Wilmington, MA, USA). cDNA was synthesized using the SuperScript III First-Strand Synthesis SuperMix (cat. no. 18080400, Invitrogen, Carlsbad, CA, USA), as specified by the supplier. SYBR Green Master Mix (cat. no. 4309155, Applied Biosystems, Foster City, CA, USA) was used as the detection method in a CFX96 Touch Real-Time PCR Detection System (cat. no. 1845097, Bio-Rad, Berkeley, CA, USA) following the manufacturer’s specifications using 100 ng of cDNA. Primer sequences used for the identification of gene cDNA fragments of *DRP1*, *FIS1*, *MFN1*, *MFN2*, *NFE2L2*, and *PPARGC1A* were previously reported by Hoffman et al., 2023 [35]; and for *SIRT1* and *ACTB* were designed (*SIRT1*: Forward 5′-TGC CGG AAA CAA TAC CTC CAC-3′ and Reverse 5′-ATG AAA CAG ACA CCC CAG CTC-3′; *ACTB*: Forward 5′-AGC CTT CCT TCC TGG GCA T-3′; Reverse 5′-CTG TGT TGG CGT ACA GGT CT-3′). Relative quantification was performed with the ∆∆Ct method.

### 2.8. Assay of SIRT1 Enzyme Activity

SIRT1 enzyme activity was measured using the commercially available Fluor de Lys SIRT1 fluorometric drug discovery assay kit (AK-555, Enzo Life Sciences, Farmingdale, NY, USA), which uses a fluorescent derivative of the peptide substrate p53-AMC, a known SIRT1 substrate. Protein extracts were obtained from untreated HTR-8/SVneo cells using NETN lysis buffer (Tris-HCl pH 7.4, 20 mM, NaCl 100 mM, EDTA 1 mM, IGEPAL 0.5%) supplemented with NaF 5mM, Nicotinamide 5 mM, β-glycerophosphate 50 mM, and protease inhibitor cocktail 1.4 mg/mL. The protein extracts were treated as previously reported, incubating for 10 min at 37 °C to allow degradation of any contaminant NAD+, and 10 mM DTT before another incubation for 10 min at 37 °C [36]. 30 μg of the protein extract was incubated for 30 min at 37 °C, adding DMSO, MEHP (5 and 200 μM) or the SIRT1 inhibitor Ex527 (100 μM), as well as the Fluor de Lys SIRT1 substrate and NAD+, both in saturated conditions, 100 μM and 0.3 mM, respectively. The enzymatic reaction was terminated by incubating the plate for 1 h at 37°C, adding Fluor de Lys Developer and nicotinamide (2 mM). Plates were read in a BioTek Synergy HTX (BioTek Instruments, Winooski, VT, USA) with an excitation wavelength of 360 nm and an emission wavelength of 460 nm. Blank relative fluorescence units (RFU) values (reactions without NAD+) were subtracted from the RFU from each independent reaction (*n* = 3), which were normalized to the control and presented as a percentage.

## 3. Results

### 3.1. MEHP Reduces Cell Viability in HTR-8/SVneo Cells

**Figure 1:**
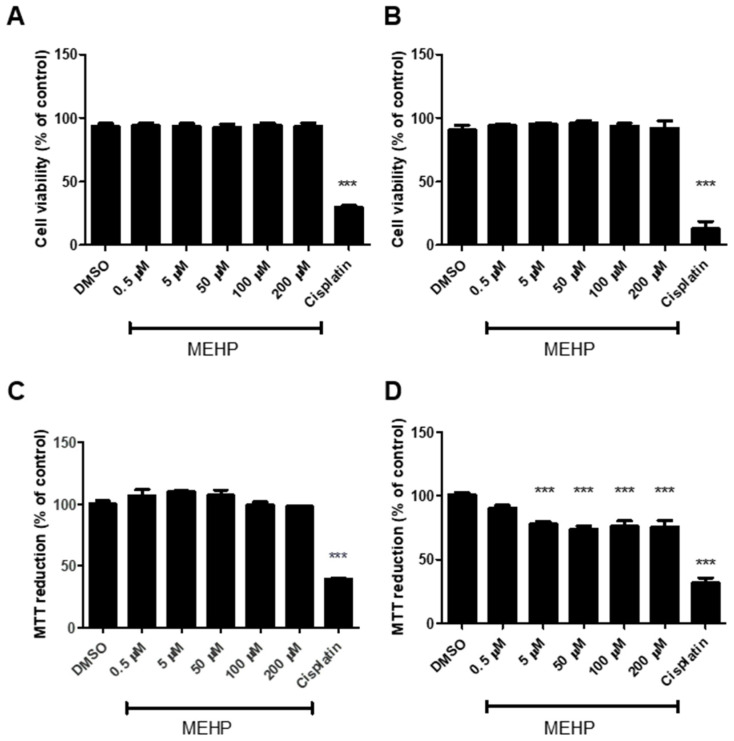
MEHP decreases MTT-based cell viability without affecting membrane integrity in HTR-8/SVneo cells. The percentage of cell viability of HTR-8/SVneo cells treated with MEHP (0.5–200 μM) at 24 h (**A**) and 48 h (**B**), determined by flow cytometry using Fixable Viability Dye eFluor 506 and the percentage of MTT redacted at 24 h (**C**) and 48 h (**D**), are presented as mean ± SE (*n* = 5). Cisplatin 1 mg/mL was used as a positive control for cell death. *** *p* ≤ 0.001.

Initially, the effects of MEHP on cell viability after 24 and 48 h were explored. This was conducted through two complementary approaches targeting different aspects of cell integrity. Assessment of membrane integrity using Fixable Viability Dye eFluor 506 (FVD, eBioscience, Carlsbad, CA, USA) showed no significant increase in cell death across the tested MEHP concentrations, with values comparable to vehicle controls (DMSO) at 24 and 48 h (Figure 1A,B).

In contrast, the MTT assay showed a significant decrease of approximately 20% of MTT-based cell viability assay after exposure to MEHP (5–200 µM; 48 h) compared to the control group (DMSO) or lower concentrations (0.5 µM) (Figure 1D). Based on the cell viability assays, we selected 5 μM and 200 μM MEHP for subsequent experiments. Although both concentrations induced comparable effects on viability, 200 μM was the highest concentration evaluated, and 5 μM was the lowest with a detectable biological response. This selection enabled the investigation of potential dose-dependent molecular effects beyond cytotoxicity, particularly to identify early mitochondrial responses that may not translate into immediate loss of viability.

### 3.2. MEHP Disrupts Mitochondrial Activity and Promotes Oxidative Stress

**Figure 2:**
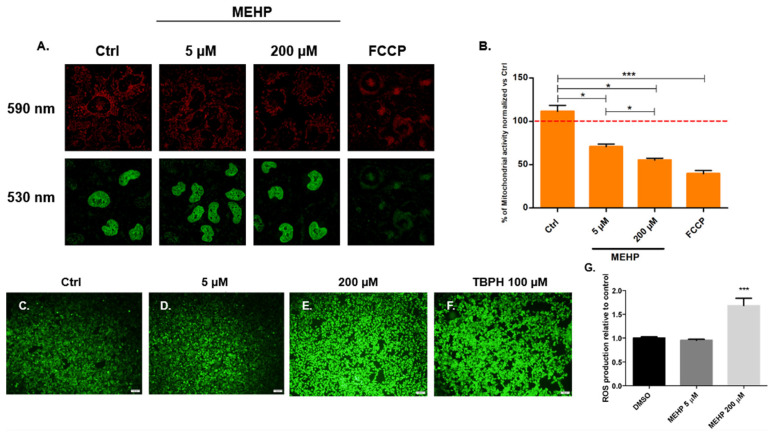
MEHP impairs mitochondrial activity and induces oxidative stress concomitantly in HTR-8/Svneo cells. (**A**) Images from confocal microscopy in both emission wavelengths, JC-1 monomers (530 nm) and JC-1 aggregates (590 nm) in HTR-8/SVneo cells exposed to MEHP (5 μM or 200 μM) and FCCP (100 μM). (**B**) Percentage of mitochondrial activity in HTR-8/SVneo cells exposed to MEHP (5 μM or 200 μM) and FCCP (100 μM), 590/530 ratio from JC1; the red dashed line indicates 100% mitochondrial activity in the control against which normalization was performed (**C**–**F**) Images from stained cells with DCFDA allow visualization of ROS in HTR-8/SVneo cells exposed to MEHP (5 μM or 200 μM) and TBPH (100 μM) as positive ROS production control. (**G**) Relative ROS quantification to the control from arbitrary fluorescence units. Results are presented as mean ± SE (*n* = 3). * *p* ≤ 0.05, *** *p* ≤ 0.001.

Considering that MEHP decreases HTR-8/SVneo MTT-based cell viability, we aimed to further investigate the effect of MEHP on mitochondrial function. The results from the JC-1 assay demonstrated (Figure 2A–F) that both concentrations of MEHP (5 μM and 200 μM) decreased red fluorescence capture at 590 nm, indicating mitochondrial dysfunction. Interestingly, this effect is enhanced at 200 μM of MEHP, suggesting a dose-dependent response. Additionally, since mitochondria are the main organelle involved in ROS production, we aimed to understand if the impairment of mitochondrial activity also affects ROS production. Notably, only the high concentration (200 μM) of MEHP shows a significant increase in ROS production in comparison to both the control (DMSO) and the low concentration (5 μM) of MEHP. These results suggest that MEHP induces mitochondrial impairment, which may result in oxidative stress in HTR-8/SVneo cells, particularly at high concentrations.

### 3.3. MEHP Induces Dose-Dependent Changes in Mitochondrial Dynamics Gene Expression

**Figure 3:**
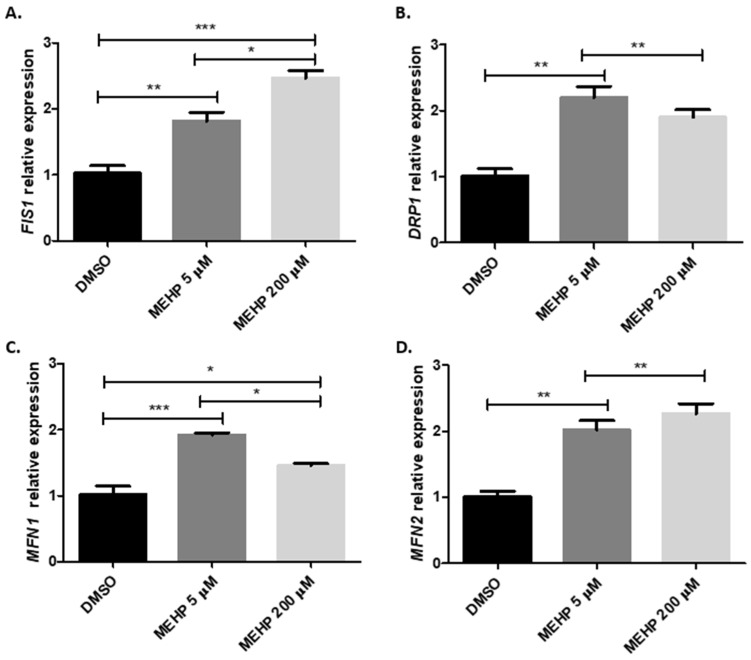
MEHP induces mitochondrial dynamics-related genes in a dose-dependent manner in HTR-8/SVneo cells. Relative expression levels of FIS1 (**A**), DRP1 (**B**), MFN1 (**C**), and MFN2 (**D**) are shown for HTR-8/Svneo cells treated with DMSO (control) and MEHP at 5 and 200 μM. Data were obtained using the ∆∆Ct method, with normalization of mRNA levels to ACTB expression. Data were analyzed by one-way ANOVA followed by the Tukey post hoc test. Results are expressed as mean ± SE. * *p* ≤ 0.05, ** *p* ≤ 0.01, *** *p* ≤ 0.001.

Having demonstrated that MEHP impairs mitochondrial activity, and to better understand the mechanisms involved, we explored whether MEHP also induces alterations in mitochondrial dynamics. Thus, the effect of both concentrations of MEHP on the expression of genes involved in mitochondrial fission (*DRP1* and *FIS1*) and fusion (*MFN1* and *MFN2*) was analyzed. As depicted in Figure 3, the results demonstrate that cells exposed to both MEHP concentrations showed a significantly increased expression of *MFN1* and *MFN2* compared to the control (Figure 3A,B). Nevertheless, the upregulation of *MFN1* was significantly higher in the low concentrations of MEHP compared to the high concentration, suggesting that such a concentration of MEHP mainly stimulates mitochondrial fusion. On the other hand, both fission genes (*DRP1* and *FIS1*) were also upregulated by both concentrations of MEHP; however, the expression of *FIS1* is significantly more upregulated in HTR-8/SVneo cells exposed to 200 μM than in the low concentration, suggesting that high concentrations of MEHP may stimulate mitochondrial fusion over fission.

### 3.4. MEHP Alters the Regulation of Mitochondrial Biogenesis in a Dose-Dependent Manner in HTR-8/SVneo Cells

**Figure 4:**
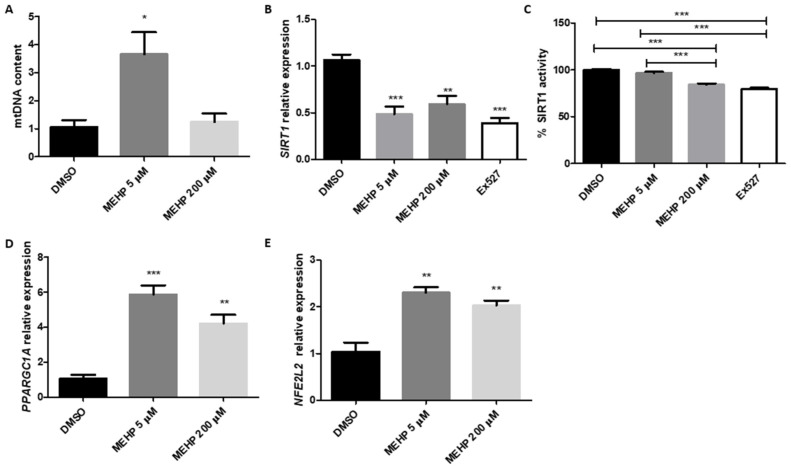
MEHP modulates mitochondrial biogenesis markers and SIRT1 activity in a dose-dependent manner in HTR-8/SVneo cells. Relative mtDNA levels using *MT-ND1* (**A**) and relative expression of *SIRT1* (**B**), *PPARGC1A* (**D**), and *NFEL2* (**E**) are shown for HTR-8/Svneo cells treated with DMSO (control) and MEHP at 5 and 200 μM. Data were obtained using the ∆∆Ct method, normalizing mtDNA levels with a sequence from the *GAPDH* promoter and mRNA levels with *ACTB* expression. The percentage of in vitro SIRT1 activity (**C**) is presented relative to the control. Data were analyzed by one-way ANOVA followed by the Tukey post hoc test. Results are expressed as mean ± SE (*n* = 3). * *p* ≤ 0.05, ** *p* ≤ 0.01, *** *p* ≤ 0.001.

Finally, to obtain a more complete overview of the MEHP effect on mitochondrial homeostasis, we also analyzed its impact on mitochondrial biogenesis (Figure 4). The results demonstrate that both concentrations impair several mitochondrial biogenesis mechanisms. HTR-8/SVneo cells exposed to MEHP 5 μM exhibited significantly more mtDNA content compared to the control and to the high concentration of MEHP (Figure 4A), suggesting that HTR-8/SVneo cells may respond to stress by inducing mitochondrial biogenesis at low concentrations of MEHP to counteract it; however, the damage at higher MEHP concentrations, such as 200 μM, may be too severe to induce a compensatory response. 

Both concentrations of MEHP upregulate the expression of the mitochondrial biogenesis regulators *PPARGC1A* and *NFE2L2* and downregulate the expression of *SIRT1* (Figure 4B–D). In vitro determination of SIRT1 activity showed a significant decrease in the presence of 200 μM MEHP, compared to the control group and the 5 μM MEHP group. However, there were no significant differences in comparison to the SIRT1 inhibitor Ex527, suggesting that high concentrations of MEHP inhibit SIRT1 enzymatic activity. This is consistent with the higher degree of alterations in mitochondrial activity (Figure 2) and the lack of an adaptive response, as reflected by the unaltered mtDNA content (Figure 4A). Overall, these results demonstrate that higher concentrations of MEHP exert detrimental effects on mitochondrial biogenesis, while HTR-8/SVneo cells exposed to MEHP 5 μM may trigger mechanisms to overcome MEHP’s impairments on mitochondrial homeostasis.

## 4. Discussion

Exposure to environmental contaminants such as MEHP has been associated with adverse pregnancy outcomes [6,15,16,17,18]. However, the mechanisms by which MEHP may compromise placental cells at the mitochondrial level remain poorly understood. In this study, we demonstrate that exposure to MEHP at concentrations encompassing reported placental concentrations and estimated plasma concentrations in populations with high exposure to MEHP leads to alterations in mitochondrial homeostasis in human trophoblast derived cells HTR-8/SVneo, as evidenced by disruption in mitochondrial dynamics, biogenesis, and activity. These findings reveal that MEHP induces a dose-dependent mitochondrial response characterized by impaired mitochondrial activity, elevated ROS production, increased expression of genes associated with fusion or fission processes, changes in mitochondrial DNA content, increased expression of genes related to mitochondrial biogenesis, and impaired SIRT1 activity. Collectively, these results suggest a compromise of mitochondrial regulation in human placental cells, which may contribute to the understanding of how environmental contaminants such as MEHP can disrupt placental cell function at the mitochondrial level, providing new insights into their role in placental pathophysiology.

While previous studies have documented decreased viability of HTR-8/SVneo cells following MEHP (180 μM, 48 h) exposure, assessed by intracellular protease activity [19]. In this study, we observed that MEHP exposure (5–200 μM, 48 h) significantly reduced MTT-based cell viability assay of trophoblast cells, which is indicative of a compromising effect on metabolic and/or redox capacity [37], yet without detectable changes in membrane integrity (Figure 1). Our data emphasizes the need to interpret viability assays in the context of their methodological differences, as these methods are not mutually exclusive, but rather provide complementary information on the cytotoxic effects of MEHP. From a cellular functional standpoint, these results suggest that MEHP can cause early toxic effects that compromise metabolic and/or redox capacity. Nevertheless, this does not appear to be sufficient to trigger late damage effects such as decreased cytoplasmic membrane integrity.

Notably, the concentrations at which these effects were observed span the levels reported in human biomonitoring studies. MEHP concentration in placental tissue in the CANDLE study has been reported to be approximately 20.4 μM [30]; meanwhile, the plasma concentrations estimated from the urine of a population of Mexican pregnant women with remarkably high levels of MEHP using high-throughput toxicokinetic models range from 7.3 to 182.9 μM [38]. These findings underscore the physiological relevance of the experimental conditions employed and the necessity of evaluating MEHP-induced alterations at environmentally realistic exposure levels. This includes concentrations that represent levels observed in populations with high and average exposure to this compound.

In line with the alterations observed in the MTT assay, which indirectly reflect impaired mitochondrial activity, further exploration of the mechanisms underlying these alterations was undertaken by assessing mitochondrial function and ROS production. Our findings demonstrated that MEHP induces a dose-dependent reduction in mitochondrial membrane potential (Δψm) and enhances ROS production, both established hallmarks of mitochondrial dysfunction (Figure 2). Notably, previous studies in HTR-8/SVneo cells have reported similar effects of MEHP diminishing Δψm (100–180 μM) [29] and significantly increasing ROS levels (45–180 μM) [19]. However, this study extends these observations by demonstrating that lower concentrations of MEHP reported by biomonitoring studies in humans (as low as 5 μM) are sufficient to impair the Δψm, highlighting the vulnerability of trophoblast cells to environmental exposure to low doses of MEHP. These findings, together with the observed enzymatic decline in the MTT assay, support the hypothesis that MEHP induces mitochondrial damage accompanied by oxidative stress, which may contribute to downstream cellular dysfunction in a dose-dependent manner.

The observed dose-dependent loss of Δψm and increase in ROS levels suggest an early mitochondrial dysfunction that could trigger adaptive or compensatory mechanisms to overcome these impairments. Mitochondrial dynamics, encompassing fusion and fission events, are pivotal in preserving mitochondrial integrity under stress conditions [39]. In this context, and despite the fact that both MEHP concentrations induced the expression of fusion and fission-related genes, the significantly higher upregulation of *MFN1* at lower MEHP concentrations (5 µM) may reflect a pro-fusion response aimed at preserving mitochondrial function [40]. Conversely, the marked induction of *FIS1* at elevated concentrations (200 µM) may indicate a transition toward mitochondrial fragmentation, a process frequently linked to severe mitochondrial stress and mitophagy to clear damaged mitochondria [40,41]. These transcriptional changes are consistent with the functional alterations in membrane potential and ROS, thereby reinforcing the hypothesis that MEHP disrupts mitochondrial homeostasis and suggesting an important role for dynamic remodeling in this process. While these findings suggest an effect on mitochondrial dynamics, these processes are tightly regulated at the post-translational level, particularly through phosphorylation-dependent control of key proteins such as DRP1 and MFN1/2; therefore, further studies are needed to evaluate total protein levels and activation states [42,43,44]. Additionally, visualization and quantification of mitochondrial network morphology (e.g., fragmentation, elongation, branching) are essential to confirm the functional implications of the observed transcriptional changes [45].

Alongside mitochondrial remodeling through fusion and fission, biogenesis pathways represent another key adaptive mechanism frequently activated under stress conditions to restore mitochondrial integrity [46]. Consistent with this notion, our results showed that both low (5 µM) and high (200 µM) concentrations of MEHP induced the expression of *PPARGC1A* and *NFE2L2*, two key regulators of mitochondrial biogenesis, suggesting the activation of a transcriptional program aimed at restoring mitochondrial homeostasis [47,48]. However, only the lower concentration (5 µM) led to a measurable increase in mtDNA content. This discrepancy suggests that while both doses trigger a compensatory biogenesis response, the process is only executed effectively under moderate stress. At higher concentrations, the lack of an mtDNA increase, despite gene upregulation, may reflect either a breakdown in downstream biogenetic processes or an inability to preserve mitochondrial genome integrity, potentially due to elevated oxidative stress, as indicated by the increase in ROS at this concentration. A similar phenomenon has been described in models of severe mitochondrial stress, such as high-dose ionizing radiation and polycyclic aromatic hydrocarbons [49,50]. The impact of low concentrations of MEHP on mtDNA content in HTR-8/Svneo cells may have been previously overlooked, as earlier studies did not examine these exposure levels, despite their detection at such concentrations in human placenta or their estimation in plasma based on urinary measurements [6,30], and reported no changes in mtDNA after a 48 h exposure to MEHP (50–180 µM) [29]. These findings underscore the importance of incorporating biomonitoring-reported concentrations when modeling MEHP exposure in vitro.

Consistent with this, *SIRT1*, a key regulator of PGC-1α-mediated mitochondrial biogenesis and a redox-sensitive deacetylase, exhibited a dose-dependent decline in both mRNA expression and enzymatic activity [51,52]. Given the role of SIRT1 in sensing energy and redox state, its impairment at higher doses of MEHP may contribute to the failure of mitochondrial biogenesis, thereby contributing to mitochondrial dysfunction under more severe stress conditions.

This study demonstrates that MEHP impairs mitochondrial homeostasis in the human trophoblast cells HTR-8/Svneo through a series of coordinated mechanisms, including impaired membrane potential, elevated oxidative stress, altered fusion–fission dynamics, and disrupted biogenesis pathways. Notably, a subset of these alterations was triggered by low MEHP concentrations (5 µM), which aligns with the range reported in human biological samples, including placental tissue and plasma estimates, and which effects were previously unexplored. This result highlights the vulnerability of trophoblastic cell mitochondria to a wide range of realistic MEHP concentrations to which people may be exposed in their environment. The observed convergence of these mitochondrial impairments suggests a potential mechanism by which MEHP could compromise placental function, hence supporting existing epidemiological associations with adverse pregnancy outcomes. However, the present study was conducted using an in vitro human-derived trophoblast cell line model, which, despite its utility in deciphering cellular and molecular mechanisms, lacks the complexity of the human placental microenvironment. The absence of maternal–fetal interactions, hormonal cues, and immune modulation may restrict the extrapolation of these findings to a physiological context. Therefore, while these data provide valuable insights into MEHP-mediated mitochondrial alterations in trophoblasts, their functional and physiological relevance should be interpreted cautiously. Additional studies using complementary models and multi-level validation are warranted to strengthen these observations and their long-term impact on placental physiology and fetal development.

## 5. Conclusions

This study provides evidence that MEHP, at concentrations of human exposure, disrupts mitochondrial homeostasis in human trophoblast HTR-8/Svneo cells by impairing mitochondrial function, altering fusion–fission dynamics, and dysregulating biogenesis. Notably, multiple mitochondrial perturbations were observed at low micromolar concentrations, emphasizing the vulnerability of placental mitochondria to environmentally detected human exposure levels. These findings offer mechanistic insight into how MEHP may interfere with placental function and contribute to pregnancy-related complications. Further in vivo and translational studies are necessary to ascertain the pathophysiological implications within the context of human gestation.

## Data Availability

The original contributions presented in this study are included in the article. Data is available on request from the corresponding author.
